# Arabidopsis Heat Shock Granules exhibit dynamic cellular behavior and can form in response to protein misfolding in the absence of elevated temperatures

**DOI:** 10.17912/micropub.biology.000285

**Published:** 2020-07-29

**Authors:** Rosalie Lawrence, Nick Kaplinsky

**Affiliations:** 1 Swarthmore College

**Figure 1. Heat shock granule dynamics in Arabidopsis roots.**
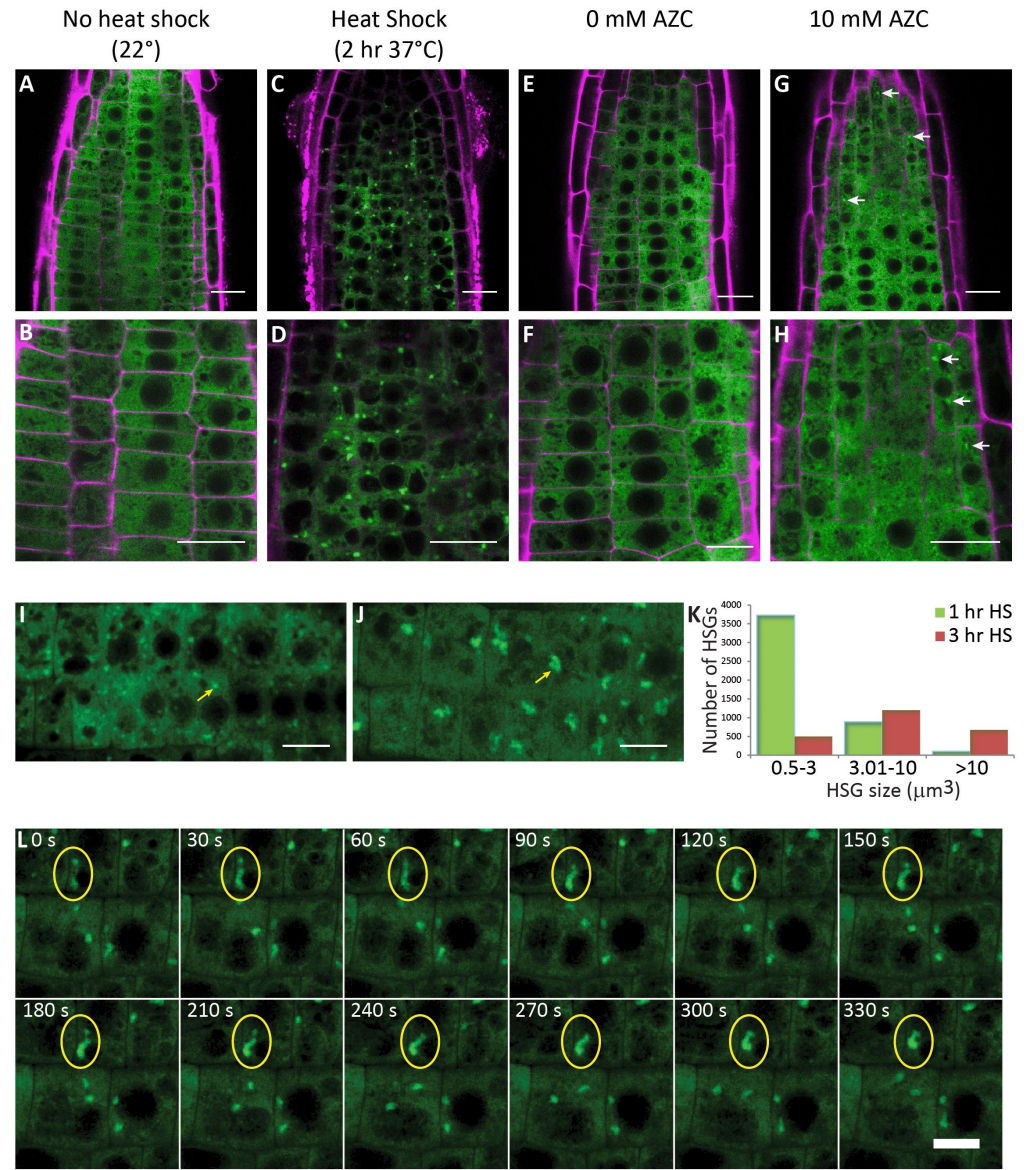
For A-H cell walls were stained with propidium iodide and are shown in magenta. A, B) *BOB1:GFP* distribution under control (22^o^C) conditions. C, D) *BOB1:GFP* distribution after a 2 hour 37^o^C heat shock. E, F) *BOB1:GFP* distribution after a 4 hour incubation in 0 mM amino acid analog L-Azetidine 2-Carboxylic Acid (AZC) at 22^o^C. G, H) *BOB1:GFP* distribution after a 4 hour incubation in 10 mM AZC at 22^o^C. Arrows highlight heat shock granules (HSGs). I) Maximal intensity projection of *BOB1:GFP* in a root tip after a 1 hour 37^o^C heat shock. J) Maximal intensity projection of *BOB1:GFP* in a root tip after a 3 hour 37^o^C heat shock. K) The number of HSGs detected in three volume categories for each heat shock (HS) duration treatment group. 1 hr HS data was collected between hours 1 and 2 of 37^o^C HS, 3 hr HS data was collected between hours 3 and 4 of 37^o^C HS. L) Maximal intensity projections of Z stacks from consecutive frames of a time lapse series of *BOB1:GFP* root tips after 2.5 hours of incubation at 37^o^C. Stacks were collected every 30 seconds. HSGs indicated by yellow oval fuse during the course of this time interval. HSG fusion was verified by examining 3D projections rotated 90° (see Movie 1). Scale bars A, C, E, G 15μm. B, D, F, H-J, L 10μm. Movie 1: Maximal Z and Y projections of a *BOB1:GFP* time lapse movie with frames obtained every 30 seconds for a total of 50 minutes. The time lapse displays root cells between 144 and 194 minutes of HS treatment at 37^o^C. Yellow ovals indicate two granules that fuse over the course of this movie. Scale bar 10μm.

## Description

In response to a heat shock (HS), plants, like all eukaryotic organisms, exhibit a highly conserved and coordinated cellular program called the heat shock response (HSR). The HSR includes the expression of genes involved in protecting cells from the deleterious effects of high temperatures. In addition, HS leads to the formation of non-membrane-bound assemblies of proteins and RNAs that play important roles in responding to cellular stress (Wallace *et al.*, 2015). Molecular chaperones known as heat shock proteins (HSPs) are expressed during a HSR. Small HSPs (sHSPs) produced during the HSR bind to misfolded proteins and prevent their irreversible aggregation while other families of HSPs facilitate the refolding of misfolded proteins in an ATP dependent manner (Richter *et al.*, 2010; Haslbeck and Vierling, 2015)**.**

Heat shock factors (HSFs) are transcription factors that directly regulate the HSR. Under non-stressful conditions, HSFs are inactivated by their interactions with cytoplasmic HSPs such as Hsp70. Conditions that result in the accumulation of misfolded proteins in the cytoplasm result in titration of the HSPs away from HSFs. Once released, HSFs can enter the nucleus, bind to DNA, and activatethe expression of HSR genes. This mechanism means that the HSR, including the production of sHSPs, can be induced by the accumulation of misfolded proteins in the absence of heat (Zheng *et al.*, 2016; Trotter *et al.*, 2002; Sugio *et al.*, 2009).

Sub-cellular structural responses to HS include the formation of cytoplasmic heat shock granules (HSGs). These granules have been reported to contain sHSPs, Hsp70s, and heat shock factors and are assumed to be sites of misfolded protein/chaperone interactions (Nover *et al.*, 1983; Perez *et al.*, 2009; Richter *et al.*, 2010; Scharf *et al.*, 1998; Siddique *et al.*, 2008; Wallace *et al.*, 2015). HSGs were thought to be distinct from stress granules (SGs) and P-bodies, sites of translational inhibition and RNA degradation, respectively (Weber *et al.*, 2008). However, recent reports suggest that HSGs share some components with SGs and that these granules may exist amongst a spectrum of non-membranous assemblies in the cell (McLoughlin *et al.*, 2016).

Here, we contribute insights into the formation of HSGs in the absence of HS. As many stresses other than HS result in protein misfolding and HSP expression in Arabidopsis (Liu *et al.*, 2011), we asked whether high temperatures are necessary for HSG formation or if the presence of misfolded proteins in the absence of HS is sufficient for HSG formation. The proline analog AZC has been shown to induce protein misfolding in the cytoplasm (Sugio *et al.*, 2009; Trotter *et al.*, 2002). We used a GFP fusion of the sHSP *BOB1* (At5g53400) to label HSGs in living cells (Perez *et al.*, 2009) to determine if treatment with AZC is sufficient for HSG formation. *BOB1:GFP* is homogenously distributed in the cytoplasm in plant cells grown at 22^o^C (Fig. 1A-B, E-F). HSG formation is evident after either a 2 hour HS at 37^o^C or after treating plants for 4 hours with 10mM AZC (Fig. 1 C-D, G-H). This result demonstrates that HSG formation does not require elevated temperatures.

We observed variations in the size and number of HSGs depending on the HS regimen used in our experiments. Longer periods of HS appeared to result in fewer and larger granules. To the best of our knowledge, no quantitative characterization of plant HSG dynamics has been published although a similar observation has been made using *eIF4A2:GFP* labeled SGs (Hamada *et al.*, 2018). We set out to ask how HSG size and number change with increasing durations of HS. In order to quantitate these differences we used 3D confocal imaging to quantitate HSG number and size during an extended 37^o^C HS. Over the course of the HS the number of HSGs decreased while their volume increased (chi-squared p<10^-175^) (Fig. 1 I-K).

Several mechanisms could explain this result. It is possible that during an extended HS a subset of HSGs grow larger while others disassemble. The oligomerization state of sHSPs is known to be dynamic and so perhaps HSGs compete with each other for misfolded protein/chaperone complexes. Alternatively, HSGs could grow over time by fusing with each other. To distinguish between these possibilities we used 4D confocal imaging to observe HSG dynamics every 30 seconds over extended periods of time (>30 minutes). We did not detect heterogeneity in HSG behavior. Rather, we repeatedly observed HSG fusion events when HSGs came into contact with each other (Fig. 1 L and Movie 1). These results suggest that during an extended HS, as misfolded proteins accumulate in the cytoplasm and start to require chaperone function, HSGs form. As the load of misfolded protein increases with extended proteostatic stress, HSGs grow larger, presumably due to the incorporation of more misfolded proteins, but also by fusing with each other.

We have demonstrated that *BOB1:GFP* can be used to visualize HSG formation caused by two different cellular stresses: HS and AZC, and that prolonged heat stress results in a decrease in the number of HSGs while their size increases. The AZC results suggest that the function of HSGs is to protect plant cells from protein misfolding, regardless of the cause of the denaturation. The ability to quantitate HSG size and number *in vivo* using *BOB1:GFP* provides a protein based marker for visualizing misfolded cytoplasmic protein accumulation in plants.

## Methods

**Plant growth and chemical treatment**

For still imaging, *BOB1:GFP* seeds (Perez *et al.*, 2009) were surface-sterilized, plated on 0.5X MS media, and grown under 16hr light/8hr dark conditions at 22°C. For heat shock treatments, plates were transferred to a 37^o^C incubator for the indicated times. Plates containing control plants were left in the 22°C incubator. For L-Azetidine-2-Carboxylic Acid (AZC) treatments, whole three-day-old seedlings grown as above were immersed in 0.5X MS media containing 10 mM AZC (BAChem, Torrence, CA). Control plants were immersed in 0.5X MS media. Both control and AZC treated plants were incubated in liquid treatment for four hours in a 22°C incubator with constant light.

For live heat shock time-lapse visualization, *BOB1:GFP* seeds were germinated in chambered coverslips (Lab-Tek, Thermo Fisher Scientific, Rochester, NY, USA) containing 4 mL 0.5X MS media and 0.5% agarose (Benchmark Scientific, Edison, NJ, USA). Coverslides were tilted 60° after seedling roots had penetrated the MS/agar layer to ensure roots would grow parallel to the coverslip. Surface-sterilized seeds were grown at 22°C in 16hr light/18hr dark conditions for 5 or 6 days.

**Imaging**

For still imaging, three day old seedlings were stained with 10 µg/mL propidium iodide to allow visualization of cell walls. Confocal imaging was performed using a Leica SP5 AOBS confocal microscope (Leica Microsystems) fitted with a heated stage (Tokai Hit, Model INUB-GSI-F1, Shizuoka-ken, Japan) to allow real-time visualization of samples during heat shock. Granule detection and quantitation was performed using Imaris 7.1 software (Bitplane Scientific, South Windsor, CT, USA). Briefly, the surfaces module of Imaris was used to automatically detect three-dimensional HSG surfaces which contained spheres of maximum diameter 0.5 µm. After automatic detection, detected surfaces were filtered based on satisfying minimum intensity threshold values. Appropriate threshold values were determined and edited by visual inspection of representative particles. Detected granule surfaces were filtered such that the minimum enclosed volume was 0.5 µm^3^.

Time-lapse series were collected for individual plant roots subjected to a continuous 37° heat shock treatment. Z-stack images of 760 X x 352 Y pixels (70 x 34 µm) with 5 Z focal planes separated by 1 µm were collected every 10 s for 50 consecutive frames (8.3 minutes).

## Reagents

*BOB1:GFP* seeds are available from the ABRC (stock number CS69998) and from NASC (stock number N69998)
